# Nursing intervention reduces depression for patients with rheumatoid arthritis: A randomized controlled trial protocol

**DOI:** 10.1097/MD.0000000000023268

**Published:** 2020-11-20

**Authors:** Moying Liu, Baoxin Shi

**Affiliations:** School of Nursing, Tianjin Medical University, Tianjin, China.

**Keywords:** depression, nursing intervention, protocol, rheumatoid arthritis

## Abstract

**Background:**

Rheumatoid arthritis (RA) is a kind of chronic disease of inflammatory joint, which can lead to the damage and disability of bone and cartilage. Psychiatric comorbidity is related to the adverse results of RA. Symptoms of depression is associated with the increased disease activity and decreased response to the treatments. Therefore, the depression may be an effective intervention target to improve the life quality and subjective health of the patients with RA. The objective of this experiment is to evaluate the effectiveness of nursing intervention for reducing depression for patients with RA.

**Method:**

It is a single-center randomized controlled study to be conducted from January 2021 to December 2021. It was admitted via the Ethics Committee of Tianjin Medical University (202018384). One hundred patients are included in the study. The inclusion criteria contains:

1. definite diagnosis of RA;
2. aged 18 to 60 years.

The exclusion criteria contains:

1. other infectious arthritis;
2. current use of oral glucocorticoids;
3. serious physical comorbidities;
4. women with the possibility of pregnancy.

All the patients participating in this study are randomly divided into control group and study group, with 50 patients in each group. The primary result is the severity of depression in the patients with RA, based on the generally utilized questionnaires (Hospital Anxiety and Depression Scale). The secondary outcome is the patients life quality, which is evaluated with the short form 36 questionnaire. The analysis of all the data are conducted with the software of IBM SPSS Statistics for Windows, version 20.

**Results:**

Table will show the clinical outcomes after various interventions.

**Conclusion:**

This paper instructs the nurses to develop protocol based on evidence to improve the clinical efficacy for the RA patients.

Trial registration number: researchregistry6114.

## 1 Introduction

Rheumatoid arthritis (RA) is a kind of chronic disease of inflammatory joint, which can lead to the damage and disability of bone and cartilage.^[1,2]^ It mainly affects joints, but it should be regarded as a syndrome involving the extraarticular manifestations, for instance, vasculitis, or pulmonary involvement, rheumatoid nodules, as well as the systemic comorbidities.^[3,4]^ The incidence rate of RA is between 0.5% and 1%, with significant declines from city to rural areas and from north to south.^[5]^ The cause of RA is still unknown, and there are no known treatments.

Psychiatric comorbidity is related to the adverse results of RA. In the RA patients, depression is related to the increased mortality, lower life quality associated with health, and higher levels of disability and pain.^[6,7]^ Depression is a predictor of the early arthritis work disability than response to treatment and disease activity. Symptoms of depression is associated with the increased disease activity and decreased response to the treatments. Treatment of depression may be the means to improve the prognosis of RA, but for patients with RA who utilize the anti-inflammatory therapy or result in symptoms exacerbations or latent adverse side effects, the generally utilized depression pharmacological treatments may have poor effect.^[8,9]^ Therefore, the depression may be an effective intervention target to improve the life quality and subjective health of the patients with RA.

Rheumatic nursing is a kind of practical specialty, which possesses a significant effect in treating the RA.^[10]^ Within a multidisciplinary team, the rheumatic nurses possess many roles, from the management of disease to coordinating the holistic care of RA patients. Nevertheless, the epidemiological surveys on the relationship between depression in patients with RA and the nursing intervention still is not clear. The objective of this experiment is to evaluate the effectiveness of nursing intervention for reducing depression for patients with RA.

## 2 Materials and methods

### 2.1 Study design

It is a randomized controlled experiment to be conducted from January 2021 to December 2021. This research is carried out on the basis of the SPIRIT Checklist of the randomized researches. It was permitted through the Ethics Committee of Tianjin Medical University (202018384), and this experiment was registered with research registry (researchregistry 6114).

### 2.2 Inclusion and exclusion criteria

One hundred patients are included in the study. The inclusion criteria contains:

1. definite diagnosis of RA;
2. aged 18 to 60 years.

The exclusion criteria contains:

1. other infectious arthritis;
2. current use of oral glucocorticoids;
3. serious physical comorbidities;
4. women with the possibility of pregnancy.

Sequence of random numbers is generated by a computer. Sequentially numbered sealed opaque envelopes are used for the concealment of random numbers. All the patients participating in this study are randomly divided into control group and study group, with 50 patients in each group.

### 2.3 Intervention

#### 2.3.1 Depression task

The social depression test is a standardized test of laboratory depression, which is composed of mental arithmetic in the front of the audience and simulated job interviews. The person implementing this test did not know the grouping assignment of participants. The total duration of this is 15 minutes, involving the interview introduction and 5-minute phase of preparation, and has been found repeatedly to induce the self-reported, the responses of autonomic nervous and neuroendocrine system.

#### 2.3.2 Depression management training

In study group, patients are intervened by 2 nurses and a therapist with an intermediate professional qualification. They are given the management of individual training on depression, focusing on the application of relaxation principles and psychological education, involving differential, cue controlled and progressive relaxation. Furthermore, teaching the patients breathing, and then giving them the visualization exercises. Patients participate in 4 separate 1-hour sessions with an experienced therapist for 2 consecutive weeks. Patients are given an MP3-player with the exercises of relaxation and, after finishing each session, the training manual involving the information presented in the session and a summary of the techniques in reducing depression. As the consolidating tasks, in their daily life, the patients participating in the study evaluate the behaviors and situations associated with depression, and then during the 14-days management intervention of depression, conduct 90-minutes relaxation exercises at least twice a day. Afterwards, in the period of 2-month follow-up, doctors encourage patients to continue their homework assignments, to conduct their relaxation exercises, and then to focus on the long-term goals. After discharge, the nurse will listen to the complaints of patients in time, and communicate with the patients along with their families regularly, and then actively deal with the related problems of patients. Control group only receive the routine nursing care in hospital.

#### 2.3.3 Outcome measures

The primary result is the severity of depression in the patients with RA, based on the generally utilized questionnaires (Hospital Anxiety and Depression Scale).^[11]^ The secondary outcome is the patients life quality, which is evaluated with the short form 36 questionnaire.

### 2.4 Statistical analysis

The analysis of all the data are conducted with the software of IBM SPSS Statistics for Windows, version 20 (IBM Corp., Armonk, NY, USA). Subsequently, all data obtained are expressed with proper characteristics, for instance, median, mean, percentage and standard deviation. Continuous and categorical variables are analyzed using χ^2^-tests and independent *t* tests, respectively. Intention-to-treat analysis is used for the outcome assessments. When *P* value <.05, it is considered to be significant in statistics.

## 3 Results

Table 1 will show the clinical outcomes after various interventions.

**Table 1:** Comparison of clinical outcomes after treatment.

Variables	Study group (n = 50)	Control group (n = 50)	*P* value
Severity of depression			
Hospital Anxiety and Depression Scale			
Short form 36 questionnaire			
Knee range of motion			
Pain score			
Medical cost			
Complications			

## 4 Discussion

RA is a kind of chronic progressive disease, which needs continuous pharmacological treatment adjustments and lifelong monitoring.^[12,13]^ Most patients will experience the unpredictable periods, they will suffer from joint pain and joint swelling, gradually leading to psychological distress, fatigue, joint damage, and a variety of daily work problems. Depression has been developed into one of the most familiar psychological problems in patients with RA.^[14,15]^ The long-term prognosis of patients with depressive RA is poor, involving the increased complications, pain as well as mortality. The published articles have reported that the depression is very common in RA and is related to poor prognosis.^[16]^ This indicates that the best care for the RA patients may involve the depression detection and treatment. Nevertheless, the results of epidemiological investigation on the relationship between depression in patients with RA and nursing intervention are mixed. These factors involve the selection bias, high levels of data loss, and the lack of statistical control over other risk factors such as the gender and age of patients, and duration of intervention.^[17]^ At the same time, the evidence that patients benefit from the rheumatic care is still increasing. This is the first randomized controlled experiment to evaluate the effectiveness of nursing intervention in the reduction of depression in RA patients. To evaluate the effect of the nursing intervention on psychological problems, a longer intervention time should be adopted.

## 5 Conclusion

This paper instructs the nurses to develop protocol based on evidence to improve the clinical efficacy for the RA patients.

## Author contributions

Baoxin Shi plans the study design, reviews the protocol and edits language. Moying Liu collects data and writes the manuscript. All authors approve the submission.

**Conceptualization:** Moying Liu.

**Data curation:** Moying Liu.

**Formal analysis:** Moying Liu.

**Funding acquisition:** Baoxin Shi.

**Investigation:** Moying Liu.

**Writing – original draft:** Moying Liu.
